# Probiotics and food allergy

**DOI:** 10.1186/1824-7288-39-47

**Published:** 2013-07-29

**Authors:** Anna Maria Castellazzi, Chiara Valsecchi, Silvia Caimmi, Amelia Licari, Alessia Marseglia, Maria Chiara Leoni, Davide Caimmi, Michele Miraglia del Giudice, Salvatore Leonardi, Mario La Rosa, Gian Luigi Marseglia

**Affiliations:** 1 Department of Pediatrics, Foundation IRCCS Policlinico San Matteo, University of Pavia, Pavia, Italy; 2 Department of Pediatrics, Second University of Naples, Naples, Italy; 3 Department of Medical and Pediatric Science, University of Catania, Catania, Italy

**Keywords:** Food allergy, Probiotics, Allergic disease, Intestinal microbiota, Children

## Abstract

The exact prevalence of food allergy in the general population is unknown, but almost 12% of pediatric population refers a suspicion of food allergy. IgE mediated reactions to food are actually the best-characterized types of allergy, and they might be particularly harmful especially in children. According to the “hygiene hypothesis” low or no exposure to exogenous antigens in early life may increase the risk of allergic diseases by both delaying the development of the immune tolerance and limiting the Th2/Th1 switch. The critical role of intestinal microbiota in the development of immune tolerance improved recently the interest on probiotics, prebiotics, antioxidants, polyunsaturated fatty acid, folate and vitamins, which seem to have positive effects on the immune functions.

Probiotics consist in bacteria or yeast, able to re-colonize and restore microflora symbiosis in intestinal tract. One of the most important characteristics of probiotics is their safety for human health. Thanks to their ability to adhere to intestinal epithelial cells and to modulate and stabilize the composition of gut microflora, probiotics bacteria may play an important role in the regulation of intestinal and systemic immunity. They actually seem capable of restoring the intestinal microbic equilibrium and modulating the activation of immune cells.

Several studies have been recently conducted on the role of probiotics in preventing and/or treating allergic disorders, but the results are often quite contradictory, probably because of the heterogeneity of strains, the duration of therapy and the doses administered to patients. Therefore, new studies are needed in order to clarify the functions and the utility of probiotics in food allergies and ion other types of allergic disorders.

## Introduction

Food allergy is an immunological disease that may have a high impact on the quality of life of both patients and their relatives, with economic consequences for the patients and the NHS (National Health Service). Even if the exact prevalence is not known, almost 20% of population refers several symptoms that can be related to food allergy, and most of them end up eliminating some food from the diet with a loss of nutritional balance.

Such a situation may become particularly harmful especially in pediatric patients, because they might experience several clinical symptoms, but also because the restricted diet, recommended until the allergy work up is completed, could lead to possible nutritional deficiencies.

In recent years many studies focused on the comprehension of molecular and immunological mechanism of allergic response and, in particular, it has been demonstrated the importance of gut microbiota for the maintenance not only of intestinal physiology, but also for the correct development of the immune system and the induction of oral tolerance.

Growing evidence indicates that dietary factors as probiotics, prebiotics, antioxidants, polyunsaturated fatty acid, folate and vitamins have positive effects on the immune functions. In particular probiotic strains show the ability to restore intestinal microbic equilibrium and modulate activation of immune cells and can explain the gradually diffusion of probiotics in clinical practice and in allergy treatment.

### Food hypersensitivity

The term “food hypersensitivity” is referred to any pathological manifestation related to food ingestion: these reactions can be mainly divided into “food intolerances” and “food allergies”, according to the molecular mechanism of action.

IgE mediated reactions are actually the best-characterized types of allergy; non-IgE mediated reactions or cell mediated responses include those reactions in which specific cells, different from mastocytes (and basophils), are responsible for the allergic reaction, and they mostly involve the gastrointestinal tract; mixed IgE mediated and cell mediated responses are those reactions in which both IgEs and immune cells are involved [1-4].

Other pathological conditions associated with symptoms similar to food allergy include pseudo-food allergy, in which, even though the clinical picture is the same that can be detected in allergic hypersensitivities, no immunological mechanism can be demonstrated.

The exact prevalence of food allergy in the general population is yet unknown. According to different meta-analysis [5,6], almost 12% of pediatric population refers a suspicion of food allergy and in Italy, about 10% of parents believe that their children suffer from food allergy [7]. However, the prevalence of food allergy is about 3% when the diagnosis is based on oral food challenge, that is the “gold standard [8,9].

Recent studies showed that, in the United States, the prevalence of food allergy is of almost 5% of children younger than 3, while the disease affects almost 4% of the adult population. Recently, Sicherer and Sampson showed a different prevalence of food allergy, stating it might interest up to 6-10% of the general population, with differences due to geographic distribution, age and ethnicity [10].

In Europe, the prevalence of food allergy is 1-2% in the adult population and 5-8% in children, even if these data are underestimated, because of the lack of good quality publications and of the different “in vitro” and “in vivo” diagnostic approaches of the different studies. Moreover, even thought the EAACI ha tried to standardize the allergy work-up for food allergies, challenges differ from one center to another, and many different specialists (pediatricians, allergists, gastroenterologists, dermatologists) seem to show different approaches to the disease.

The other datum that has to be considered as clearly established, is that the prevalence of food allergy is higher during the first years of life, with an estimated incidence between 6 and 8% before the age of 2. Clinicians may therefore assist to a progressive decrease of food allergies over time, as patients grow up.

The increasing immigration in Italy of children coming from different countries has brought up the issue on whether they might change the prevalence of the disease in our Country. Nevertheless, a retrospective study conducted between 1999 and 2001 in 23 Italian Pediatric Clinics in Italy showed that children of immigrant families who were born in Italy or who arrived in Italy during the first years of life, show the same incidence of food allergy as Italian children, with, therefore, an increase of the overall prevalence [11].

Most of cutaneus allergic reactions to food are due to the role of allergen specific IgE antibodies with their high affinity receptors (FceRI), expressed on mast cells and basophils, and low affinity receptors (FceRII), expressed on macrophages, monocytes, lymphocytes and platelets. The linkage of IgE with FceRI leads to receptor crosslinking and release of cellular mediators [12,13].

The amount of potentially dangerous foods is quite low (egg, milk, peanut, tree nuts, fish, shellfish, wheat and soy) and their allergenicity is due to the their proteic component. The “major” allergenic segments or “epitopes” of these proteins are water-soluble glycoproteins with a molecular weight usually comprised between 10 and 70 Kd and a good resistance to heat and acid and proteasic digestion. In addition, the presence of immunostimulatory factors in the food may also contribute to such a sensitization [14,15].

Usually, only the minority of subjects exposed to a food allergen develops an allergic response. In fact, the list of food mentioned above includes products that are commonly ingested by almost the entire population, while allergic symptoms appear only in a small percentage of persons. The biochemical characteristics of food allergens cannot indeed explain alone its allergenicity, but the allergic response is also due to specific genetic or epigenetic characteristics of the subject. Even though no specific genes responsible of allergic reactions have been firmly identified so far, it is widely accepted, in clinical practice, that atopy (defined as a personal or familial tendency to produce IgE antibodies in response to low doses of allergens) [1] relates to a certain predisposition to develop food allergy as well. The natural response after exposure to new food allergens is known as tolerance.

IgE mediated allergic reactions are characterized by several different clinical manifestations that may involve any kind of organ and develop few minutes or few hours after food ingestion (usually within 2 hours). Acute systemic reactions are caused by the activation of mastocytes and basophils and consequent production of chemical mediators. Oropharyngeal symptoms are usually represented by labial and palatal tinging and itching, angioedema, hoarseness, dysphonia, dry cough. Gastrointestinal symptoms include nausea, vomit, cramps, colic and diarrhoea. Other symptoms may be lacrimation and nasal congestion, urticaria, other cutaneous manifestations, bronchospasm [16] and even anaphylaxis [16,17].

Non-IgE mediated food allergy is less common and the cellular mechanisms involved in this type of hypersensitivity are not completely understood yet. Clinical manifestation are mainly characterized by acute and chronic inflammation of the digestive tract or of the skin that seem to result from an eosinophilic and T cell response directly towards allergenic proteins, leading to the release of chemical mediators that drive the inflammatory response, with the involvement of specific cytokines. These reactions have often a hereditary and ethnic component, with a predisposition in caucasian male subjects [1]. Clinical manifestation of non-IgE mediated disorders include eosinophilic esophagitis; dietary-protein induced enterocolitis (diarrhea, emesis, lethargy, poor growth), that usually arises during childhood and later undergoes resolution, and is often caused by milk, soy and rice; dietary-induced proctitis (typical of childhood), characterized by bleeding and mucilage in stools and often associated to vaccine milk ingestion, besides several other rare forms.

Some disorders are associated with a mixed IgE-/cell-mediated reactions, such as atopic dermatitis, eosinophilic gastroenteritis and eosinophilic esophagitis.

Oral tolerance is established in early childhood and causes a suppression of local and systemic immune response against food allergens and bacteria of endogenous intestinal microflora. The organism may develop an effective immune response against foreign pathogens, but no response is activated towards self-antigens. This condition, defined as “specific immunologic hyporesposiveness”, depends on an intact and immunological active gastrointestinal barrier [1,18,19].

This barrier includes the epithelial cells joined by tight junctions, mucosal layer, lumenal and brush border enzymes, bile salts and extreme pH levels. Moreover, intestinal microflora plays a central role in the regulation of gastrointestinal stability and physiology and in the manteinance of an integrity of permeability of intestinal mucosa. The increased permeability of intestinal mucosa and early exposure to allergenic antigens have been proposed as a possible cause of sensitization in infants. The allergic response in sensitized subjects is a consequence of the loss of oral tolerance and of the regulation of the T cell response [18,20].

### Intestinal microflora and allergy

It is widely accepted that early exposure to environmental allergens may influence the maturation of the immune system and the possible predisposition to develop immune-mediated disorders such as autoimmune or allergic disease [20].

The intestine, besides its digestive and absorbent activities, has a key role in the immune system as well. Part of the intestinal barrier is composed by bacterial microflora, which promotes intestinal physiology and provides protection against external pathogens. Moreover, the gut microflora is the most important source of microbial stimulation and it plays a central role for the maturation of the immune system and for the maintenance of gut homeostasis.

The human gastrointestinal tract (GI), at birth, is sterile and the immune system is naїve. Soon after birth, GI tract is colonised by numerous types of microorganisms, and after approximately one week after birth the colonisation is complete. Intestinal epithelium in mammalian, performs its physiological functions in a microbe rich environment, while the microbes improve host defences.

Human intestinal micro flora is composed by Bacteroides, Clostridia, Enterobacteria, Bifidobacteria and Lactobacilli, with significant differences between breast-fed and formula-fed newborns.

The postnatal period of a new human being is characterized, from the microbiological point of view, by the formation of a new ecosystem: the microflora of the human gut. While in adulthood a number of barriers exert a potent selective action on bacteria arriving from the mouth, in very first stage of life these barriers are at low levels and feeding (breast vs bottle feeding) play a key role in determining the microflora composition.

According to data obtained by means of classical microbiological techniques, bifidobacteria, lactobacilli, and other anaerobic bacteria appears to reach the gut after 2–3 days.

An additional source of bacteria for the breast-fed neonates is mother’s milk, which contains up to 10^9^ microbes/L in healthy mothers. The most frequently encountered bacterial groups include staphylococci, streptococci, corynebacteria, lactobacilli, micrococci, propionibacteria and bifidobacteria, originating from the nipple and surrounding skin as well as the milk ducts in the breast. Particularly it has even suggested that human breast milk is a relevant source of lactobacilli for newborn, overall L.acidophilus followed by L.casei and L.paracasei, whereas babies with a standard formula harboured mainly L.delbruekii and L.reuteri, L.acidophilus however is also present in these babies but at lower level.. In adult subjects, GI tract is colonised by over 10^14^ microorganisms, and, so far, less then 50% of the species of the gut micro flora have been identified [20-22].

The interaction between microbiota and host organism can be either symbiotic or commensal. The bacteria of microbiota can facilitate both the absorption of nutrients and the prevention of intestinal colonization by pathogenic microrganisms.

Commensal microorganisms, acquired since the first postnatal period, are required for the development of immune tolerance, not only towards themselves, but also to other antigens, such as food antigens. The interaction with the commensal microflora of the GI tract is one of the environmental signals that support T-cells (mainly Th1) maturation. The tolerance to the microbiota is supported by the ability of commensal bacteria to suppress inflammatory response (i.e. by down regulating the NK-kB activity) and by absence of virulence factors expressed by commensal bacteria that can be recognized by Toll-like receptors (TLRs) on the surface of immune system cells [23,24].

Over these past few years, industrialized countries have outpointed a significant increase in autoimmune and allergic diseases, which are considered to be related by Th1- and Th2-mediated mechanisms, respectively. The increase in immunologically mediated disorders might be explained by an impaired maturation of the immune function during the early stages of life, with a consequent loss of immune tolerance. This mechanism seems to be largely mediated by the microenvironment of the intestinal mucosa and adjacent lymphoid tissues.

According to the “hygiene hypothesis” low or no exposure to exogenous antigens in early life may increase the risk of allergic diseases by both delaying the development of the immune tolerance and limiting the Th2/Th1 switch. An altered microbial flora seems to allow the persistence of Th2 cytokines (IL4, IL13, IL5), predominant at birth, and forbid the shift towards a predominant Th1response, with production of IFN-g and IL12. However allergic diseases (Th1-mediated) and autoimmune diseases (Th2-mediated), such as diabetes mellitus type 1 are not mutually exclusive [25]. This may suggest that the hygiene hypothesis may oversimplified the immunologic basis of allergic diseases.

The importance of commensal bacteria could also be highlighted by the recent “old friends hypothesis” (a revised version of the “hygiene hypothesis”), which claims that the presence of these bacteria is crucial for the maturation of regulatory dendritic cells, that promote T regulatory cells (T_regs_) differentiation through TGF-b and IL-10 production. This mechanism is related to a suppression of the inflammatory response against commensal bacterial antigens, which then leads to the maturation of an high number of regulatory dendritic cells, to the processing of self or food antigens and to the induction of immunological tolerance [26,27].

Recent studies on cell-based mechanisms of autoimmune and allergic diseases have led to the discovery of another T cell subset, Th17, which seems to have a role in the pathogenesis of allergic diseases. IL-17 levels are increased in asthmatic patients sputum and may contribute to the pathogenesis of non-atopic and/or non-eosinophil/neutrophil-dominant asthma [28-30]. Changes in the symbiotic microflora may have a role in balance between inflammatory Th17 cells and T_regs_ Foxp3+ cells operating in the intestine [31].

The importance of microbiota for the regulation of immune responses is supported by the observations of different composition of intestinal microflora between allergic and non-allergic children. Björksten et al., showed a high prevalence of *Staphylococcus Aureus* and a lower percentage of Bacteroides and Bifidobacteria in allergic children at the age of 2 [32]. Another study has later confirmed these results and showed a higher prevalence of Clostridia in allergic children [33].

Early in life the immune system is immature and requires stimuli from the environment, such as microbial exposures, to mature properly. The gut microbiota composition has previously been reported to differ during infancy prior to the development of allergic disease, implying a role of the gut microbiota in promoting tolerance to harmless antigens through education of the immune system. Bifidobacteria have been demonstrated to have a species-specific influence on gut immunity, and thus the early composition of bifidobacteria may have a major impact on the naïve immune system. It has been clearly demonstrate that breast-milk contains bifidobacteria and that a constant supply of bifidobacteria to the infant’s intestine is thus assured during breastfeeding. Allergic infants have indeed been found to be colonized by bifidobacteria less often and with lower concentrations. It is also noteworthy that *B*. *adolescentis* is found more often in the intestinal microbiota in allergic than in non-allergic children.

More recently, the bifidobacterial predominance in the intestinal microbiota of breast fed infants has been linked to the direct transfer of maternal bifidobacteria to newborns in breast-milk and consequently in promoting a healthy intestinal microbiota, reducing the risk of atopic diseases.

Even if there are many unclear aspects on cellular and molecular interaction between the intestinal microflora and the immune system, the importance of a correct homeostasis of microbiota for reducing the risk to develop an allergic disorder is widely accepted and the possible use of probiotic strains, as a support therapy, is a central argument in the scientific debate.

### Probiotics

The critical role of intestinal microbiota in the development of immune tolerance improved recently the interest on dietary supplements, as probiotics, that can promote intestinal colonization and gut homeostasis.

The term “probiotic” derives from the Latin word “pro” and the Greek word “bios,” meaning “for life”, and is related to the concept of “probiotic”, probably introduced by Elie Metchnikoff in 1907. He proposed the idea that ingestion of live microbes could have beneficial effects on human health. The definition of Metchnikoff includes both the use of a food matrix fermented by beneficial bacterium and a “concentrated” bacterial supplementation in the diet.

The definition of “probiotics” evolved over the years and in 2001 a group of experts convened the currently FAO/WHO definition of probiotics as “live microrganisms which, when administered in adequate amounts, confer a health benefit on the host” [34]. Similarly, the Italian Ministry of Health defined probiotics as “microrganisms which, once ingested in adequate amounts, have beneficial effects on the organism” [35].

The main characteristic of probiotics is the human origin of these bacterial strains, that is one of the criteria for their selection. Moreover, to define a good probiotic, several requirements are needed, such as: the ability to adhere to gut epithelium cells, to exclude or reduce pathogenic adherence, to persist, multiply and produce acids, hydrogen peroxide and bacteriocins against pathogenic growth. They are considered safe, non-invasive, non-carcinogenic and non-pathogenic and are able to aggregate to form a normal balanced microflora [36].

Probiotics consist in bacteria or yeast, able to re-colonize and restore microflora symbiosis in intestinal tract. Probiotic bacteria usually belong mainly to *Lactobacillus* and *Bifidobacterium* groups, in particular *Lactobacillus acidophilus* and *Bifidobacterium bifidus*, which include different strains (*L*. *rhamonusus*, *L*. *bulgaricus*, *L*. *salivarius*, *L*. *plantarum*, *L*. *casei*, *B*. *infantis*, *B*. *longum*, *Streptococcus thermophilus*). Some common probiotics, as *Saccharomyces boulardii*, are yeasts [37]. Several bacterial strains are acid and bile tolerant and can be isolated from the mammal gastrointestinal system.

Many positive effects have been attributed to probiotics strains as an improvement of the GI tract homeostasis and of digestion, a regularization of the intestinal bowel and a possible modulation of the immune system.

#### Safety of probiotics

One of the most important characteristics of probiotics is their safety for human health, that is one of the crucial points for their selection. In particular, the characterization of a probiotic strain is based on the absence of resistance to clinical or veterinary antibiobiotics as well as the absence of virulence factors [38].

The Scientific Committee on Animal Nutrition of the EU (SCAN), the EFSA panel on additives, products and substances used in animal feed (FEEDAP) and the Italian Ministry of Health recommended the verification of the absence of transferable antibiotic resistant genes as a prerequisite for approval of a microrganism [39,40].

The identification of bacterial strain is necessary not only for safety reasons, but also to prove their efficacy due to the fact that different strains of the same species may exert different effects on the host [27].

The consumption of probiotics increased in recent years and they are considered as “generally regarded as safe” (GRAS), but many studies showed controversial results about their safety and their efficacy in clinical practice.

The possible complications related to use of probiotics could be the development of bacteriemia, sepsis or endocarditis, the toxicity and the metabolic effects on the GI tract and the possibility of transfer antibiotic resistance to the GI flora, even if bacteriemia and fungemia may occur rarely and are often related to the use of saprophytic probiotics [38]. Moreover, some probiotics bacteria showed negative characteristics as the presence of virulence factors, possible acquisition of virulence genes or antimicrobial resistance that can induce unwanted resistance in endogenous bacterial populations [39].

Some probiotics strains, such as *Lactobacillus*, *Leuconostoc*, *Enterococcus* and *Bifidobacterium*, have been isolated from infected sites leading to the problem of a possible translocation of these probiotics. Liong underlined that probiotics translocation is a rare event in healthy humans, but the possible damaging effects of probiotics translocation can occur in immunocompromised patients [40].

Several studies regarding the use of probiotics in critical patients showed controversial results and further investigation are required to better understand the mechanisms of probiotic translocation and infection.

#### Probiotics and immunity

Thanks to their ability to adhere to intestinal epithelial cells (IELs) and to modulate and stabilize the composition of gut microflora, probiotics bacteria may play an important role in the regulation of intestinal and systemic immunity. Probiotics may influence the functionality of dendritic cells (DCs), NK cells, monocytes, macrophages and, to a lesser extent, B cells.

The adhesion of some probiotics, in particular lactic acid bacteria (LABs), to the epithelial intestinal wall promote their capture in the Peyer’s patches, where they directly modulate both activation and proliferation of DCs. Probiotics are able to stimulate both IL-10 and IL-12 production by myeloid DC (mDCs), that promote Th1-Th2/Treg polarization, and TNF-a and IL-6 by DCs. Several LABs also induce DCs maturation through the induction of IL-12 and TNF-a production and promote IFN-a production by plasmacytoid DCs (pDCs). Mature DCs are potent activators of NK cells, through cytokines production, as IL-12 and IL-15, that induce NK cell activation, proliferation and cytotoxic activity. Some probiotics strains are also able to stimulate monocytes to produce IL-12 and NK cells for the production of IFN-g [41-43].

The ability of LABs to shape NK/DCs crosstalk and to directly stimulate NK activation highlights the importance of some probiotics strains in promoting NK-dependent IFN-g production, and thereby Th1 polarization. The presence of LABs in early life may be useful to promote Th1 polarization and prevent Th2-mediated disorders.

Probiotics play their immunomodulatory activity either after recognition of the whole bacterial cell or of bacterial components eg. PPRs by several surface receptors expressed on innate and acquired immune cells, called toll-like receptors (TLRs). These active molecules are DNA and cell wall components, such as unmethylated CpG DNA (ligand of TLR9), peptidoglycan, lipoteichoic acid and lipopolysaccharides (ligands of TLR2, TLR1 and TLR4). The activation of TLRs, after recognition of bacterial antigens, leads to cell maturation, and cytokine production and proliferation [43-45].

The activation of DCs promotes T cells response and IL-10 and IL-12 production, making a linkage between microbiota, innate immunity and adaptative immunity. Specific probiotics strains may induce directly a B cells response, increasing humoral immunity, and T cells response, increasing cell-mediated immunity. In particular, in the intestinal *lamina propria*, B cells differentiate in plasma cells producing dimeric IgA antibodies. Secretory IgA are released into the intestinal lumen, where they have a crucial role in the mucosal immunity, after the binding with a specific transporter receptor on basolateral surface of intestinal epithelial cells [46,47]. An experimental study showed that the administration of a mixture of *Lactobacillus casei*, *L*. *acidophilus*, *L*. *rhamnosus*, *L*. *delbrueckii* subsp. *bulgaricus*, *Streptococcus salivarius* subsp. *thermophilus* and *Lactococcus lactis* had positive effects on the respiratory system by preventing and reducing respiratory infections due to an increase of IgA-secreting cells in bronchial mucosa in mice [48].

### Probiotics and allergy

It is well known that the intestinal mucosa microenvironment plays a crucial role in the maturation of the immune system since birth. In particular, the balance between Th1 and Th2 immune response and the induction of immune tolerance is regulated in the gut by the interaction of microflora bacterial antigens and immune cells.

The conclusion that changes in the composition of the gut microbiota are implicated in the pathogenesis of allergic disorders has increased the attention of researchers on the use of probiotics in order to treat and/or prevent allergic diseases [49,50]. Clinical studies on allergic patients yielded to controversial results over the efficacy of probiotics as an anti-allergic treatment.

Many studies focused on the use of probiotics strains for atopic dermatitis and other cutaneous allergic manifestations. The disruption of the skin barrier initiates the subsequent atopic march that might lead to allergic airways diseases: in fact, children affected by atopic dermatitis are more prone to develop allergic asthma [51-54].

The first report by Majama et al., showed a modest reduction of atopic dermatitis in children after one month of treatment with *L*. *rhamnosus* and a concomitant reduction of TNF-a and a1-AT in feaces of children after probiotic consumption, when compared to the placebo group [55]. However, no effect was detected regarding systemic immune responses [56].

The idea of a strict correlation between the composition of intestinal microflora and the predisposition of allergic diseases is supported by several observations of changes in intestinal microbiota of atopic children with a high prevalence of Clostridia [57-59]. These studies reinforce the hypothesis of a role of probiotics in the recurrence of allergic respiratory symptoms in children. An Italian study conducted in 2007 on a cohort of 187 pre-school children (119 with asthma, 131 with allergic rhinitis and 63 with both) with allergic symptoms to airborne allergens showed that the consumption of fermented milk containing *L*.*casei* DN-114 001 reduced of 33% the recurrence of yearly rhinitis with a two times lower incidence in children receiving probiotics compared to the placebo group. Moreover, the genetic analysis of intestinal microflora performed in a subgroup of 45 patients showed a high prevalence of *L*.*casei* DN-114 001 and other probiotic strains in the patients group compared to controls; the colonization of the GI tract persisted for 6–12 months in almost all subjects [60].

The different composition of the intestinal microflora in allergic children suggests that the immediate modification of post-natal colonization could be useful to prevent allergic diseases. Kalliomäki et al., proposed the administration of probiotics during pregnancy and in the early post-natal period. They reported a reduction of 50% of infantile eczema [61]. The same group also reported that the administration of *Lactobacillus GG* to both mothers and infants during the firs 6 months *post partum* had positive effects in preventing the appearance of early atopic diseases in children at high risk, with a marked reduction of asthma and eczema, if compared to the placebo group [62].

This study paved the way to many subsequent researches on the use of different probiotic strains to treat and prevent allergic diseases, but, unfortunately, the results seem controversial and inconsistent [63]. In particular, Kopp et al. reported that *Lactobacillus* GG supplementation in pregnant women and early infants was not effective in reducing either the incidence of atopic dermatitis or the severity of the disease in affected children, but it was associated with an increased incidence of episodes of wheezing [64].

Moreover, a recent meta-analysis made a comparison between several studies conducted from 2001 and 2009 on probiotic consumption during pregnancy and early life. The meta-analysis showed that administration of lactobacilli in pregnant women could be useful in preventing atopic dermatitis in children from 2 to 7 years of age [65], while a mixture of various probiotic strains does not affect the development of atopic dermatitis [66-70].

The effects of a mixture of different probiotic strains have been investigated by Kukkonen et al. in a randomized double blind controlled trial in a cohort of pregnant women, who assumed during 2 to 4 weeks before delivering a mixture of *Lactobacillus rhamnosus* GG(ATCC 53103), *L rhamnosus* LC705 (DSM 7061), *Bifidobacterium breve* Bb99(DSM 13692), and *Propionibacterium freudenreichii* ssp. *shermanii* JS(DSM 7076) containing galacto-oligosaccharides (GOS). Probiotic treatment did not reduce the incidence of IgE-associated diseases in children by the age 2 years, but significantly prevented the development of atopic dermatitis [71].

Another recent study demonstrated the absence of efficacy of *Lactobacillus rhamnosus* to change the composition of gut microflora. The consumption of a probiotc preparation by 50 women during late pregnancy did not affect the composition of intestinal microflora of infants after 7 days of breast feeding, compared to control group [72].

The usefulness of probiotics not only for prevention, but also as a therapeutic support for allergy is not completely demonstrated, and the results remain controversial. Many studies focused on the use probiotics in the treatment of atopic dermatitis. A randomized placebo-controlled trial showed the efficacy on adult atopic patients of *Lactobacillus salivarius* LS01 after 16 weeks of supplementation. LS01 was associated with an improvement of skin manifestations, a significant reduction of Staphylococci in feaces of the probiotic group and modulation of Th1/Th2 cytokine profile [73].

The use of probiotic strains in therapy against allergic disease has been widely investigated and it has been hypothesized that the therapeutic potential of these bacterial strains decreases with increasing age due to the completion of intestinal colonization and the establishment of allergic phenotype. A great number of studies mainly focused on therapeutic use of probiotics during the paediatric age, in particular during infancy and early childhood.

Recently the effect of supplementation of formulated milk with *Lactobacillus paracasei* CNCM I-2116 or *Bifidobacterium lactis* CNCM I-3446 has been investigated in infants at 3–6 months of age affected by atopic eczema, compared to placebo (fed with an hydrolysed whey-formula). The researchers showed no benefit in the treatment of eczema after supplementation with *L*. *paracasei* or *B*. *lactis* and no effects on progression of allergy from 1 to 3 years [74,75].

Frequently the use of combinations of probiotics and prebiotics (synbiotics) showed beneficial effects. Wu et al. reported that the supplementation with a synbiotic [*Lactobacillus salivarius* and fructo-oligosaccharide (FOS)] had positive effects in 60 paediatric atopic patients aged 2–14 years affected by severe to moderate atopic dermatitis. The group treated with the combination of *Lactobacillus salivarius* and FOS showed a significant reduction of atopic dermatitis after 8 weeks of treatment compared to the group treated with only FOS [76].

Moreover we previously evaluated the efficacy of a mixture of *Lactobacillus paracasei* I 1688, *Lactobacillus salivarius* I 1794 (PSMIX) after 30 days treatment in paediatric patients affected by atopic dermatitis. The probiotic preparation was well tolerated and induced regularization of intestinal function. Even if only one patient referred a significant improvement of atopic dermatitis, “in vitro” immunological investigations showed an increase in Th1 immune response, as in the IL-12 and IL-10 cytokine production, in CD4^+^ T cell response and in Natural Killer activity. These results confirmed a previous report on “*in vitro*” immunomodulatory effects of the two bacterial strains and their mixture in PBMCs (peripheral blood mononuclear cells) of healthy volunteers [77,78].

The efficacy of probiotics in the treatment of allergic respiratory diseases has not been completely demonstrated, due to the relative paucity of studies in this field. Asthma and respiratory allergic may occur in paediatric age, but most studies have been conducted on adults, who present a more established intestinal microflora and more established patterns of immune response; in this group, probiotics seem to be less effective [79]. In young children (6–24 months) with recurrent wheeze and an atopic family history, oral *Lactobacillus* GG supplementation had no positive effects on asthma or atopic dermatitis and only mild effects on allergic sensitization for the following 6 months [80]. A recent study with a mouse model of allergic asthma showed that oral administration of *L*. *gasseri* attenuated allergen-induced airway inflammation and induced a reduction in IL 17-mediated immune response [81].

Few studies, conducted on animal models, focused on the evaluation of the efficacy of probiotics in the prevention and treatment of food allergy, with controversial results. *Lactococcus lactis* NCC 2287 oral administration for 7 weeks in sensitized mice reduced allergic manifestation and “*in vitro*” production of IL-12, CCL11 and CCL17 in the ileum, compared to control mice [82]. An “*in vitro*” study showed that oral administration of a probiotic mixture containing 8 different strains (*Lactobacillus acidophilus*, *L*. *delbrueckii* subsp. *bulgaricus*, *L*. *casei*, *L*. *plantarum*, *Bifidobacterium longum*, *B*. *infantis*, *B*. *breve*, *Streptococcus salivarius* subsp. *thermophilus*) could have beneficial effects in ovalbumin-sensitized mice, with a reduction in symptoms severity and a down regulation of Th2 cytokine mRNA expression, in particular IL-13, IL-4 and IL-5 in the jejunum [83].

The contradictory results of the studies on probiotics efficacy in the prevention and treatment of allergy may be due to the great heterogeneity of strains, duration of therapy and doses used. Even if there are promising data on the treatment of atopic dermatitis, little is known about the efficacy of probiotics for respiratory allergic symptoms and food allergy [84,85].

## Competing interest

The authors declare no conflicts of interest for the present paper.

## Authors’ contributions

All the authors contributed to the present work. All authors read and approve the final manuscript.
